# Development of High Performance Quantum Image Algorithm on Constrained Least Squares Filtering Computation

**DOI:** 10.3390/e22111207

**Published:** 2020-10-25

**Authors:** Shumei Wang, Pengao Xu, Ruicheng Song, Peiyao Li, Hongyang Ma

**Affiliations:** Quantum Physics Laboratory, School of Sciences, Qingdao University of Technology, Qingdao 266520, China; wangshumei@qtech.edu.cn (S.W.); xupa123@163.com (P.X.); songruicheng164@163.com (R.S.); peiyao_li_1477@163.com (P.L.)

**Keywords:** quanta image computation, quantum image algorithm, image restoration, algorithm analysis

## Abstract

Recent development of computer technology may lead to the quantum image algorithms becoming a hotspot. Quantum information and computation give some advantages to our quantum image algorithms, which deal with the limited problems that cannot be solved by the original classical image algorithm. Image processing cry out for applications of quantum image. Most works on quantum images are theoretical or sometimes even unpolished, although real-world experiments in quantum computer have begun and are multiplying. However, just as the development of computer technology helped to drive the Technology Revolution, a new quantum image algorithm on constrained least squares filtering computation was proposed from quantum mechanics, quantum information, and extremely powerful computer. A quantum image representation model is introduced to construct an image model, which is then used for image processing. Prior knowledge is employed in order to reconstruct or estimate the point spread function, and a non-degenerate estimate is obtained based on the opposite processing. The fuzzy function against noises is solved using the optimal measure of smoothness. On the constraint condition, determine the minimum criterion function and estimate the original image function. For some motion blurs and some kinds of noise pollutions, such as Gaussian noises, the proposed algorithm is able to yield better recovery results. Additionally, it should be noted that, when there is a noise attack with very low noise intensity, the model based on the constrained least squares filtering can still deliver good recovery results, with strong robustness. Subsequently, discuss the simulation analysis of the complexity of implementing quantum circuits and image filtering, and demonstrate that the algorithm has a good effect on fuzzy recovery, when the noise density is small.

## 1. Introduction

Just as Feynman first discussed quantum computing in 1982 [1] and Bennett and Brassard, who performed key distribution on the quantum channel, proposed BB84 protocol [2] with modern technology. This is a new burgeoning branch of quantum physics is updating 19th-century image processing algorithm for quantum information systems at present. Some quantum information algorithms have been extensively studied in the past few decades. The quantum prime factorization algorithm that was proposed by Shor [3] and the quantum search algorithm proposed by Grover [4] have proved the computing power of quantum computers. In these case, increasing amounts of researchers have began to explore the various applications of quantum computers [5,6,7,8,9]. In addition, interrelated disciplines are also emerging. Quantum image algorithm is an interdisciplinary field in its infancy.

Physicists nowadays who work at the intersection of two fields—quantum information theory and image processing—live it. The existing computing technology is inefficient for the demand of image processing. The development of classic image processing is limited by image resolution. Because of the properties of quantum superposition and entanglement, the efficiency of algorithm can be greatly improved. It is possible to use quantum computer to solve the problem of time and space consumption in this kind of complex image processing algorithm. At first, S. E. Venegas Andraca proposed the concept of a quantum matrix shown here, exists as a thought experiment [10]. Since then, other algorithms [11,12] have been constantly improved. In previous studies, researchers have proposed many quantum image models, which play a good role in representing image information in practice. These include qubit lattice [13], entangled image [14], real ket [15], flexible representation of quantum images(FRQI) [16], and the novel enhanced quantum representation of digital images (NEQR) [17]. The image processing function [18,19,20,21] in the classical field is very powerful, and the development of quantum image is also following the direction of classical image processing. However, due to the late start, most of the existing models can only be used for simple image processing. Yuan et al. [22] proposed two kinds of improved quantum dilation and erosion operations in order to reduce the time complexity of quantum morphology operations in 2016. In this algorithm, all operations are done via quantum circuits. They constructed a system for using qubits to code and process image information. In 2020, Ma et al. [23] improved the algorithm on the basis of Yuan et al. and proposed a more consistent algorithm for quantum dilation and erosion as well as its implementation in the IBM quantum simulator. This is a powerful step forward in quantum image processing. In many digital image processing algorithms, noise cancellation is one of the basic procedures that must be considered. However, not a lot of studies have considered the noise problem in the quantum image processing mode. In 2017, Liu et al. studied restoration to remove noise in quantum images [24]. Although the filtering model of quantum image works well, the problem of blurring should also be considered. In digital image processing, we should not only consider noise, but also the deblurring problem caused by external factors. It can be said that the quality of digital images plays a crucial role in the process of communication and visual perception. High quality images will bring richer content and information, and even give the viewer a beautiful enjoyment, while poor quality images will not only lose a lot of image information, but also sometimes bring uncomfortable feelings to the viewer.

The photos taken by the camera may appear blurred due to some environmental influence factors of distance and light intensity. Shaking during filming can cause this problem. Therefore, we need to analyze this point spread function to solve the impact that is caused by the blur and restore the image. Because of the increase of image resolution, the development of traditional digital image processing have been restricted. The merge of quantum computation and traditional digital image processing methods is considered to be a promising candidate to solve the problem. Because quantum image processing will use the superposition and entanglement properties of quantum mechanics to calculate all pixels of the image at the same time, it can achieve exponential acceleration compared to classical algorithms. Accordingly, this paper proposes a quantum image restoration model that is based on constrained least squares filtering. This model can not only solve the problem that the degradation function is sensitive to noise, but also eliminate the influence of some noise.

The remaining paper are organized, as follows: Section 2 describes the NEQR model and constrained least square filtering computation. The detailed quantum image process, including NEQR construction process and basic quantum circuits, is described in Section 3. Section 4 and Section 5 describe complexity analysis and simulation experiments. Finally, in Section 6, the conclusions and prospects of future work are introduced.

## 2. Preliminaries

### 2.1. NEQR Model

In the NEQR representation model, the position information and color information of grayscale images can be represented by two entangled quantum sequences. Quantum entanglement is a quantum mechanical phenomenon, in which entangled quantum sequences have a correlation. The model uses *q* quantum bits to represent color information. *q* is the image color depth and it can represent all colors in the grayscale range $\left[0,2^{q}-1\right]$. An image compiled through the NEQR description model can be expressed, as follows:

(1) $$|I\rangle =\frac{1}{2^{n}}\sum_{i=0}^{2^{2n}-1}|c_{i}\rangle ⊗|i\rangle$$

$|c_{i}\rangle$ and |i〉 respectively represents the color image color Information and the position information of the image:

(2) $$\begin{array}{c} |c_{i}\rangle =|c_{i}^{q-1}\cdots c_{i}^{1}c_{i}^{0}\rangle ,|c_{i}^{k}\rangle \in \left\{0,1\right\},k=q-1,\cdots 1,0. \end{array}$$

(3) $$\begin{array}{c} |i\rangle =|y\rangle |x\rangle =|y_{n-1}y_{n-2}\cdots y_{0}\rangle |x_{n-1}x_{n-2}\cdots x_{0}\rangle ,|y_{i}\rangle |x_{i}\rangle \in \left\{0,1\right\}. \end{array}$$

NEQR images can be measured in order to obtain images exactly the same as the original image color information. Therefore, NEQR images are more flexible in color processing.

### 2.2. Constrained Least Square Filtering Computation

**Figure 1:**
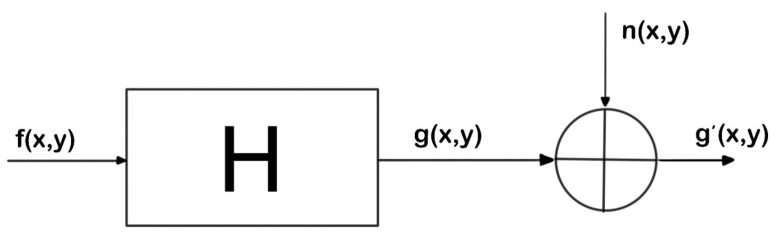
Image degradation process.

The input-output relationship of image acquisition, in Figure 1:

(4) g(x,y)=H[f(x,y)]+n(x,y)

As a significant part of digital image processing, image restoration is helpful to eliminate the distortion and deterioration in the process of image information transmission so as to obtain high-quality image information. Image restoration is an inverse process of image degradation, where *H* points to the diffusion function.

The constrained least square filtering model [25] only requires knowledge of the variance and mean of the noise signal, and the noise parameters can be inferred from the known degraded image.The key of constrained least square filtering is to overcome the relative sensitivity of noise to *H*. *H* is the entire degradation process. Image degradation can be obtained after the original image is convolved with the system. Therefore, the image restoration can achieve de-convolution operation on the output result in order to obtain the original image estimation. To obtain the degenerate image g(x,y), one needs to superimpose the original image f(x,y) and noise n(x,y) under the degenerate operator h(x,y). Accordingly, a constrained minimum criterion function *C* can be set up, as follows

(5) $$C=\sum_{0}^{M-1}\sum_{0}^{N-1}{\left[\nabla^{2}f\left(x,y\right)\right]}^{2}$$

In the constrained minimum criterion function *C*, *M* and *N*, respectively, represent the value range of pixels, $\nabla^{2}f\left(x,y\right)$ is the Laplace transform of the original image f(x,y).

The constraint is $∥G-H\hat{F}{∥}_{2}^{2}={∥N∥}_{2}^{2}$. Here, in the frequency domain, *G* is the degraded image, $\hat{F}$ is the estimate of degraded image, *N* is additive noise, and Laplace operator $\nabla^{2}$ is smoothness. To find the minimum value of the function, one needs to use the Lagrange multiplier method and convert the constraint term into the Lagrange multiplier term.

The matrix form of the Laplace operator can be expressed as:

(6) $$\nabla^{2}=\left[\begin{array}{ccc} 0 & -1 & 0\\ -1 & 4 & -1\\ 0 & -1 & 0 \end{array}\right]$$

The Laplace transform of a continuous function f(x,y) at *x* and *y* is defined as:

(7) $$\nabla^{2}f=\frac{\partial^{2}f}{\partial x^{2}}+\frac{\partial^{2}f}{\partial y^{2}}$$

For digital images, it can be obtained by difference calculation

$$\begin{array}{cc} \frac{\partial^{2}f}{\partial x^{2}} & =\frac{\partial (f(i+1,j)-f(i,j))}{\partial x}\\ \; & =f(i+1,j)-2f(i,j)+f(i-1,j) \end{array}$$

Similarly,

(8) $$\frac{\partial^{2}f}{\partial y^{2}}=f\left(i,j+1\right)-2f\left(i,j\right)+f\left(i,j-1\right)$$

Therefore, combining the second order difference of the horizontal and vertical directions of the image, the Laplace calculation method is as follows:

(9) $$\nabla^{2}f=f\left(i+1,j\right)+f\left(i-1,j\right)+f\left(i,j+1\right)+f\left(i,j-1\right)-4f\left(i,j\right)$$

Additionally likewise, use the Laplace convolution model and convert the constraint term to the Lagrange multiplier term, so the Lagrangian function $L(\hat{F},\lambda )$ is:

(10) $$L\left(\hat{F},\lambda \right)={∥P\hat{F}∥}_{2}^{2}-\lambda \left(∥G-H\hat{F}{∥}_{2}^{2}-{∥N∥}_{2}^{2}\right)$$

where *H* represents the fuzzy matrix and *P* is the Fourier transform of the Laplace operator. Different point spread function are used to restore the image.

When one take the derivative of the function $L(\hat{F},\lambda )$ with respect to $\hat{F}$, one obtains

(11) $$\begin{array}{cc} \frac{\partial L(\hat{F},\lambda )}{\partial \hat{F}} & =\frac{2\left[∥P\hat{F}{∥}_{2}^{2}+\lambda \left(114-H\hat{E}∥{}_{2}^{2}-∥{N∥}_{2}^{2}\right)\right]}{2\hat{F}}\\ \; & =2p^{*}p\hat{F}+\lambda \left(-2H^{*}G+2H^{*}H\hat{F}\right) \end{array}$$

when $\frac{\partial L(\hat{F},\lambda )}{\partial \hat{F}}=0$, one get the minimum $\hat{F}$:

(12) $$\hat{F}\left(u,v\right)=\left[\frac{H^{*}\left(u,v\right)}{{\left|H\left(u,v\right)\right|}^{2}+\gamma {\left|P\left(u,v\right)\right|}^{2}}\right]G\left(u,v\right)$$

where $\gamma =\frac{1}{\lambda }$, $H^{*}$ is the complex conjugate of *H*.

## 3. Quantum Image Algorithm

### 3.1. Initialization

For a $2^{n}\times 2^{n}$ classic image, NEQR requires a total of 2n+q qubits to save information. According to the initialization state, the quantum image can be constructed in two steps:

(1) The initial quantum state is converted to an equal weight superposition of all pixels in the image. $U_{1}=I^{⊗q}⊗H^{⊗2n}$. Hence, when $U_{1}$ applied to an initial quantum state |ψ〉, the quantum state is converted to an intermediate quantum state. This quantum evolution process can be expressed as:

(13) $$U_{1}{=(I|0\rangle )}^{⊗q}{⊗(H|0\rangle )}^{⊗2n}=\frac{1}{2^{n}}{|0\rangle }^{⊗q}⊗\sum_{i=0}^{2^{2m-1}}|i\rangle =|\psi_{1}\rangle$$

(2) Each pixel on the intermediate state is assigned a color bit. Since in the new quantum image, the pixels of the image are still processed in the superposition state, the operation in this step still needs to be divided into $2^{n}\times 2^{n}$ sub-operations in order to assign color bits to each pixel of the image. Suppose that the sub operation of each pixel is $\Omega_{YX}$, so the sub operation of each pixel of the image can be expressed as:

(14) $$U_{YX}=\left(I⊗\sum_{j=0,j\neq Y}^{2^{n}-1}\sum_{i=0,i\neq X}^{2^{n}-1}\left|ji\rangle \langle ji\right|\right)+\Omega_{YX}⊗\left|YX\rangle \langle YX\right|$$

Subsequently, when the operation $U_{YX}$ is applied to the intermediate state $|\psi_{1}\rangle$, the quantum evolution process of the whole system is:

(15) $$\begin{array}{cc} U_{YX}|\psi_{1}\rangle & =U_{YX}\left(\frac{1}{2^{n}}\sum_{j=0}^{2^{n}-1}\sum_{i=0}^{2^{n}-1}{|0\rangle }^{⊗q}\langle ji|\right)\\ \; & =\frac{1}{2^{n}}\left(\sum_{j=0}^{2^{n}-1}\sum_{i=0,ji\neq YX}^{2^{n}-1}{|0\rangle }^{⊗q}\langle ji|+|f\left(Y,X\right)\rangle |YX\rangle \right) \end{array}$$

In the classical digital image, the quantum image NEQR model is quantified and the translated data set is obtained through cyclic shift operation.

### 3.2. Quantum Circuit Implementation

We can obtain the gradient value of all pixels by quantum image cyclic shift operation. The original image $I_{xy}$, and $|I\rangle =\frac{1}{2^{n}}\sum_{Y=0}^{2^{n}-1}\sum_{X=0}^{2^{n}-1}|C_{YX}\rangle |Y\rangle |X\rangle$, then $C_{y-}$, Shift $I_{xy}$ one-unit downwards,

(16) $$I_{xy-1}=C_{y-}I_{xy}=\frac{1}{2^{n}}\sum_{Y=0}^{2^{n}-1}\sum_{X=0}^{2^{n}-1}|C_{Y-1X}\rangle |Y\rangle |X\rangle$$

The output $I_{xy-1}$, then $C_{x+}$, shift $I_{xy}$ one-unit rightwards,

(17) $$I_{x+1y}=C_{x+}I_{xy}=\frac{1}{2^{n}}\sum_{Y=0}^{2^{n}-1}\sum_{X=0}^{2^{n}-1}|C_{YX+1}\rangle |Y\rangle |X\rangle$$

The output $I_{x+1y}$, then $C_{y+}$, shift $I_{xy}$ one-unit upwards,

(18) $$I_{xy+1}=C_{y+}I_{xy}=\frac{1}{2^{n}}\sum_{Y=0}^{2^{n}-1}\sum_{X=0}^{2^{n}-1}|C_{Y+1X}\rangle |Y\rangle |X\rangle$$

The output $I_{xy+1}$, then $C_{x-}$, shift $I_{xy}$ one-unit leftwards,

(19) $$I_{x-1y}=C_{x-}I_{xy}=\frac{1}{2^{n}}\sum_{Y=0}^{2^{n}-1}\sum_{X=0}^{2^{n}-1}|C_{YX-1}\rangle |Y\rangle |X\rangle$$

**Figure 2:**
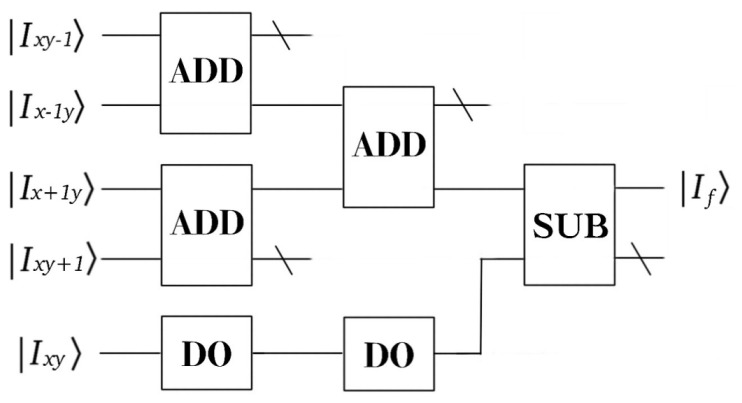
Realization of quantum circuits $U_{L}$. On the shifted image set, the gradient is calculated by addition, subtraction, and doubling.

At last, the output $I_{x-1y}$. One obtains the pixel value of the neighborhood by cycle shift operation, and then calculate the gradient according to Laplace’s template, where a quantum black box $U_{L}$ is designed to compute the gradient of the Laplace operator, in Figure 2.

### 3.3. Cycle Shift, Addition Subtraction and Double Operation

**Figure 3:**
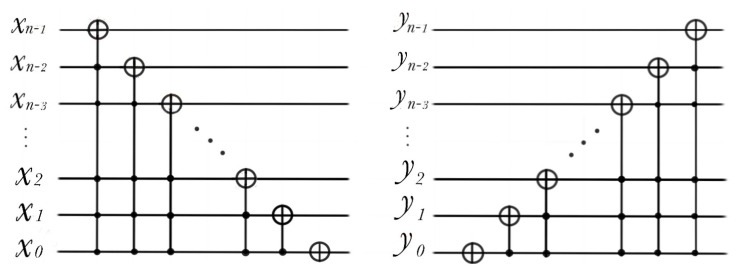
Quantum circuits of C(x+) and C(y−) for n-qubit sequence length.

Cyclic shift transform can move the whole image at the same time, and each image will get the pixel information of its field, as in Figure 3.

(20) $$C\left(x\pm \right)|I\rangle =\frac{1}{2^{n}}\sum_{Y=0}^{2^{n}-1}\sum_{X=0}^{2^{n}-1}|C_{YX^{\prime }}\rangle |Y\rangle |X\rangle$$

(21) $$C\left(y\pm \right)|I\rangle =\frac{1}{2^{n}}\sum_{Y=0}^{2^{n}-1}\sum_{X=0}^{2^{n}-1}|C_{Y^{\prime }X}\rangle |Y\rangle |X\rangle$$

For the image $I_{A}$ and $I_{B}$, the sum of the images is $I_{C}$

(22) $$|I_{C}\rangle =\frac{1}{2^{n}}\sum_{YX=0}^{2^{2n}-1}|C_{YX}\rangle |YX\rangle =\frac{1}{2^{n}}\sum_{YX=0}^{2^{2n}-1}|A_{YX}+B_{YX}\rangle |YX\rangle$$

The following is the addition operation of the quantum image, and the expression of the addition operation is:

(23) $$|I_{c}\rangle =ADD\left(I_{A},I_{B}\right)$$

**Figure 4:**
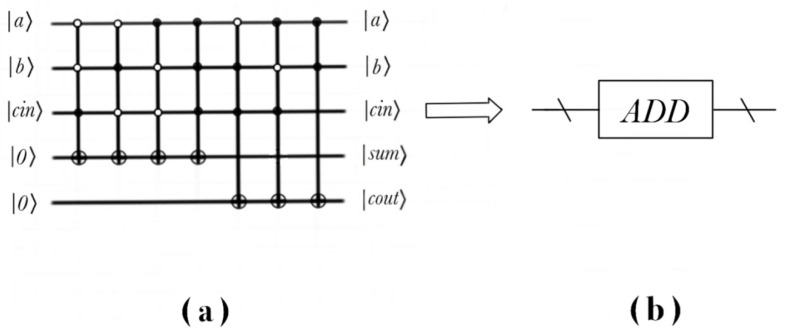
Realization of quantum circuit of adder and simplified model. (**a**) The concrete quantum circuit of adder; (**b**) Simplified model of adder.

The realization process of the quantum circuit of adder is shown in the Figure 4.

Add the pixel value to the corresponding complement in order to obtain the difference of pixel subtraction between two quantum images $|C_{YX}\rangle =|A_{YX}\rangle -|B_{YX}\rangle$.

The subtraction operation of the quantum image is deduced, as follows:

(24) $$|I_{C}\rangle =SUB\left(|I_{A}\rangle ,|I_{B}\rangle \right)$$

**Figure 5:**
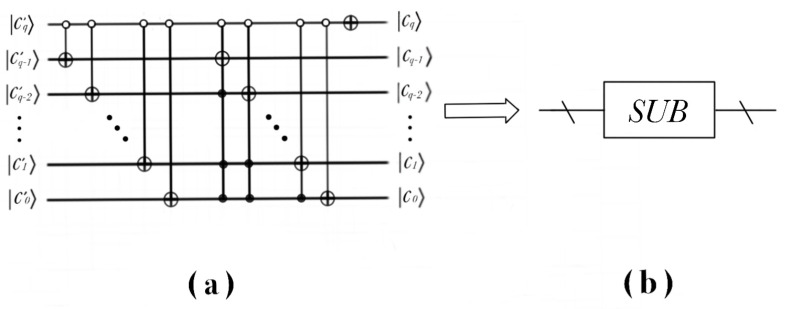
Realization of quantum circuit of subtracter and simplified model. (**a**) The concrete quantum circuit of the subtracter; (**b**) A simplified model of a subtracter.

The realization process of the quantum circuit of the subtracter is shown in the Figure 5.

**Figure 6:**
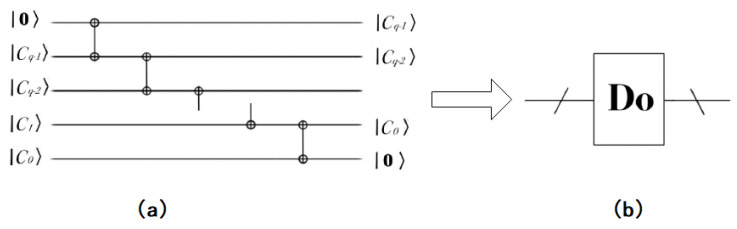
Double operation of the quantum circuit implementation and simplified model. (**a**) The concrete quantum circuit of doubling operations; (**b**) A simplified model of doubling operations.

If one wants to shift the binary sequence to the right by this, one obtains $|2\cdot C\rangle =|c_{q-1}c_{q-2}\cdots c_{1}c_{0}0\rangle$. In order to form a sequence of qubits, add an auxiliary qubit |0〉, then obtain $|0\rangle ⊗|C\rangle =|0c_{q-1}c_{q-2}\cdots c_{1}c_{0}\rangle$. The realization process of the quantum circuit of double operation is shown in the Figure 6.

### 3.4. Restoration for Deblurring of Quantum Image

If the premise of prior knowledge of noise signal *n* is lacking, then the process of image restoration is an estimation $\hat{f}$ of trying to find undegraded image *f*. This needs to make $H\hat{f}$ and *g* mean square error as small as possible. In other words, in the sense of the least mean square error, $H\hat{f}$ is closest to *g*. In order to solve this problem, Laplace operator is introduced, according to the previous discussion. The sensitivity to noise is reduced by means of smoothing measures.

If space domain recovery method is needed, then we need to solve the problem of estimating the least square of the original image *f*.

The quantum images can be obtained through the coordinate transformation. Through the cyclic shift operation of the original image $|I_{xy}\rangle$, we can obtain a set of quantum images: $|I_{xy-1}\rangle ,|I_{x-1y}\rangle ,|I_{xy}\rangle |I_{x+1}y\rangle ,|I_{xy+1}\rangle$. Next, we calculate the gradient based on the convolution template.

(25) $$\begin{array}{cc} |\mu_{1}\rangle & =qADD(|I_{xy-1}\rangle +|I_{x-1y}\rangle )\\ |\mu_{2}\rangle & =qADD(|I_{x+1y}\rangle +|I_{xy+1}\rangle )\\ |\mu_{3}\rangle & =qADD(|\mu_{1}\rangle +|\mu_{2}\rangle )\\ |\xi_{1}\rangle & =qADD(|I_{xy}\rangle +|I_{xy}\rangle )\\ |\xi_{2}\rangle & =qADD(|\xi_{1}\rangle +|\xi_{1}\rangle )\\ |\varpi \rangle & =qSUB(|\mu_{3}\rangle -|\xi_{2}\rangle ) \end{array}$$

The processed image is stored in |ϖ〉

(26) $$|I_{\hat{f}}\rangle =\frac{1}{2^{n}}\sum_{X=0}^{2^{n}-1}\sum_{Y=0}^{2^{n}-1}|f_{\varpi }\left(Y,X\right)\rangle |YX\rangle$$

Because solving the minimum value of the function consumes too many resources, so, according to Equations (11) and (12), we estimate the minimum of the function $\hat{F}$ when the function $\frac{\partial L(\hat{F},\lambda )}{\partial \hat{F}}$ is equal to 0. According to the frequency domain filtering [26], an estimated expression for an image:

(27) $$|I_{\hat{F}}\rangle =\left[\frac{H^{*}}{H^{*}H+\gamma {\left|P\right|}^{2}}\right]|I_{G}\rangle$$

$|I_{\hat{F}}\rangle$ is the Fourier transform of the estimation of degraded image. $|I_{G}\rangle$ is the Fourier transform of fuzzy image. Where γ is a parameter that can be adjusted. $H^{*}$ is the complex conjugate of *H* and it represents the Fourier transform of fuzzy matrix. It is worth noting that, if γ=0, the constrained least square filtering can be reduced to inverse filtering.

## 4. Complexity Analysis

We discuss the simulation analysis of the complexity of implementing quantum circuits and image filtering in this section. In quantum image processing, it is known that the complexity depends on the number of elementary gates in quantum image processing. Consider a digital image with a size of $2^{n}$×$2^{n}$ as an example. The complexity of the basic quantum gate includes NOT gates and controlled NOT gates, which is considered to refer to a n-controlled NOT (n-CNOT) gate that equal to 2(n-1) Toffoli gates and one CNOT gate, and one Toffoli gate can be simulated by six CNOT gates.

From the introduction of Reference [17], we know that the computation complexity of this stage does not exceed $O(qn2^{2n})$, because some operations will be performed for each pixel individually. The quantum circuit will construct the NEQR quantum image |I〉.

When an image is filtered by Laplace operator, the circuit design mainly involves two parts: shift transformation and gradient calculation of pixel points. In total, we use the 4 shift operations to get the pixel values in other locations. In addition, each shift conversion will cost $O(n^{2})$ the time complexity by the article [12]. In the light of the Laplace operator template, the complexity of quantum circuit mainly includes 5 addition operations and 1 subtraction operation, which are 8q−4 and $3q^{2}$. The cost of quantum black boxes $U_{L}$ module is $2^{q+3}-2$.

Therefore, the quantum cost of image filtering for a $2^{n}$×$2^{n}$ digital image is:

(28) $$\begin{array}{cc} \; & qn2^{2n}+4n^{2}+5\left(8q-4\right)+3q^{2}+2^{q+3}-2\\ \; & =qn2^{2n}+4n^{2}+3q^{2}+40q+2^{q+3}-22\\ \; & =O(qn2^{2n}+n^{2}+2^{q+3}) \end{array}$$

Hence, if the quantum image has been constructed, our proposed scheme can restore the quantum image that is based on NEQR, and its computational complexity of around $O(n^{2}+2^{q+3})$.

## 5. Simulation Experiment

In our simulation, the experiments were conducted on the classic computer with an Intel(R) Core(TM) i5-7500 CPU processor. The environment for the Windows 7 operating system and Matlab(2016a).

The peak signal-to-noise ratio (PSNR) is an objective criterion for evaluating images. It works by using the mean square error (MSE) between the undegraded original image *I* and the processed image *G*. In the Formula (30), MAXI is the maximum value in the pixel range.

(29) $$MSE=\sum_{j=0}^{m-1}\sum_{i=0}^{n-1}\left(\frac{{\left(I\left(i,j\right)-G\left(i,j\right)\right)}^{2}}{m*n}\right)$$

(30) $$PSNR=10\cdot \log_{10}\left(\frac{MAX_{I}^{2}}{MSE}\right)=20\cdot \log_{10}\left(\frac{MAX_{I}}{\sqrt{MSE}}\right)$$

**Figure 7:**
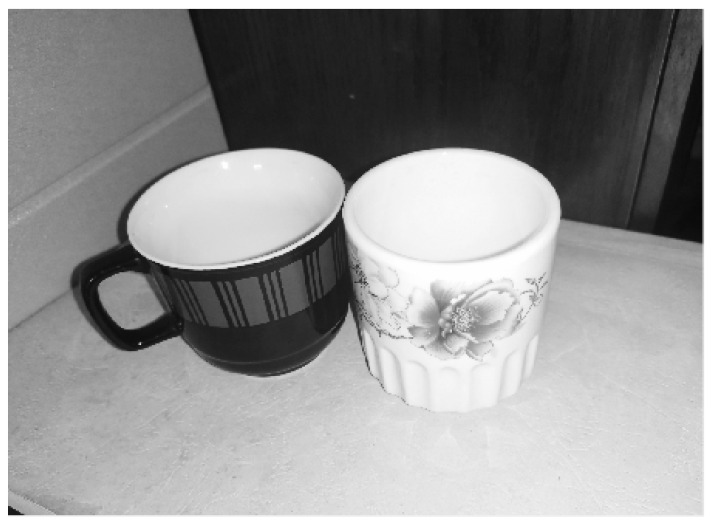
The original image.

**Figure 8:**
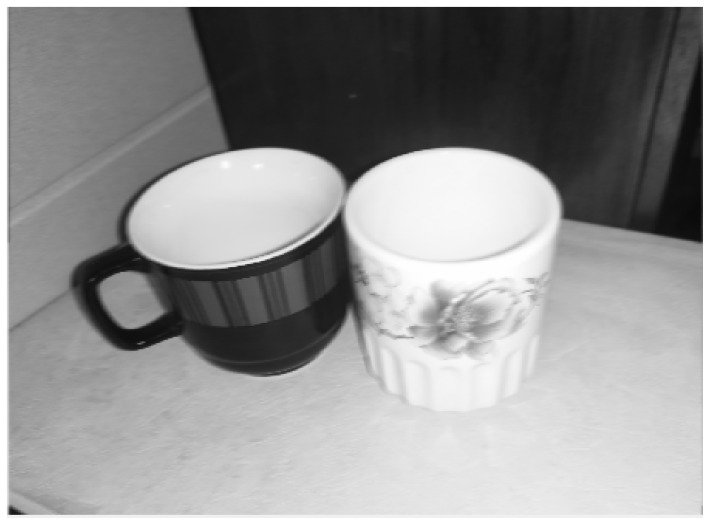
Motion blurred image.

**Figure 9:**
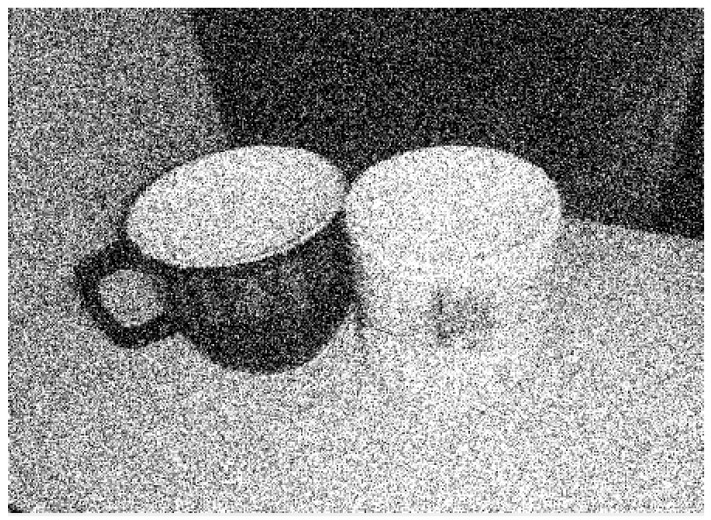
Motion blur and noise images.

**Figure 10:**
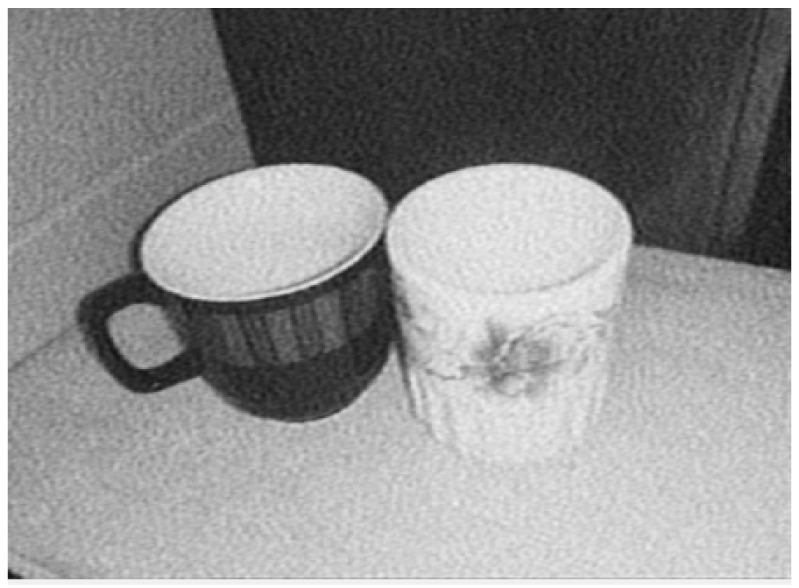
The restored image after filtering.

The original input image is shown in the Figure 7. In motion blur functions, offset Angle is 11 and the offset length is 21 (see Figure 8). Gaussian noise can be added to the fuzzy image, as well as the noise density is 0.0001 (see Figure 9). We can calculate PSNR from the Formula (30). The PSNR of the motion blur image and noise-added image are 23.1184 dB and 23.1112dB, respectively. After the model processing (Figure 10), the PSNR of the restored image is 31.6785 dB. Different restoration effects can be seen according to different parameters. When the noise density is small, the algorithm has a good effect on fuzzy restoration, as shown in the Table 1.

## 6. Conclusions and Prospects

In this paper, quantum image-deblurring algorithm for quantum image with size $2^{n}\times 2^{n}$ that is based on constrained least squares filtering is proposed and studied. The quantum circuit we designed based on Laplace operator solves the problem of image blur very well. In terms of the computational complexity, for the NEQR quantum image of size $2^{n}\times 2^{n}$, the quantum circuit that we constructed can recover the image whose computational complexity does not exceed $O(n^{2}+2^{q+3})$.

In fact, under the influence of the surrounding environment, image blurring is a problem that needs to be studied. When compared with classical computers, quantum computers have great advantages. This method can not only effectively remove the fuzzy problem, but also filter out a variety of noise. Image deblurring is always an important research direction in image processing. In the future, we will also study various deblurring problems.

## Figures and Tables

**Table 1:** Average peak signal-to-noise ratio (PSNR) on 100 images by different parameters for image restoration.

Fuzzy Factor (Angle:Length)	PSNR (Fuzzy)	Noise Density	PSNR (Noise)	PSNR (Recovery)
11:21	23.1184	0.0001	23.1112	31.6785
20:22	21.6673	0.008	18.3736	21.5656
10:20	21.8055	0.08	11.8724	21.4558
11:23	21.3195	0.1	11.1295	21.9005
